# Biomaterials‐driven stem cell therapy for tissue repair and functional rehabilitation after ischemic stroke

**DOI:** 10.1002/btm2.70060

**Published:** 2025-09-26

**Authors:** Mengjie Wang, Yuanyuan Ran, Jianshen Liang, Fanglei Li, Ning Li, Zitong Ding, Jianing Xi, Wei Su, Lin Ye, Zongjian Liu

**Affiliations:** ^1^ Beijing Rehabilitation Hospital Capital Medical University Beijing China; ^2^ Beijing Tsinghua Chang Gung Hospital School of Clinical Medicine, Tsinghua University Beijing China; ^3^ School of Materials Science and Engineering Beijing Institute of Technology Beijing China

**Keywords:** biomaterial, ischemic stroke, neurorestoration, stem cell therapy

## Abstract

Ischemic stroke is a serious cerebrovascular disease with limited effective treatments. While stem cell therapy shows promise, ensuring cell survival and integration into neural networks remains a challenge. Recent research shows tissue engineering can greatly fix these flaws. Notably, we focus on the structure–activity relationship of biomaterials. How cell behavior can be most beneficially regulated by changes in the physical structure of the cell carrier itself is certainly a new perspective for cost saving and effectiveness increasing compared to the delivery of expensive biotrophic factors. However, there is a lack of research on biomaterials applied to ischemic stroke, especially in combination with stem cells. No biomaterial has even been approved for clinical trials in stroke. We provide a systematic summary of biomaterials‐driven stem cell therapy for ischemic stroke in terms of pathomechanisms, applications, and clinical translational challenges; we attempt to build a bridge from laboratory research to clinical translation in stroke treatment.

**Translational Impact Statement**

Many biomaterials with good biocompatibility have been used in patients or are undergoing clinical trials as are stem cell transplantation clinical trials. We here summarize the synergistic cooperation between biomaterials and stem cells, hoping that this will benefit patients with stroke, especially the patients with severe cerebral ischemic stroke.

## 1 INTRODUCTION

Stroke is a severe acute cerebrovascular disease, with ischemic stroke (IS) comprising approximately 71% of cases. It is the second leading cause of death after neonatal disorders in children and ischemic heart disease in adults.^1^ The intravenous tissue plasminogen activator (IV‐tPA), approved by the US Food and Drug Administration (FDA) in 1995, remains the most effective IS treatment in recent decades. However, its narrow therapeutic time window limits clinical application, with only a small fraction of patients (~5%) ultimately benefiting from this therapy.^2^ Research into new treatments for IS is ongoing. Many promising biomolecules and drugs in laboratory studies show low clinical efficacy, long translation times, and few reach the market.^3^ The complexity of the central nervous system (CNS) and the limited regenerative capacity of the CNS contribute to these challenges. Particularly, the blood–brain barrier (BBB)—a highly selective semipermeable membrane—severely restricts drug permeability and results in poor targeting efficacy.^4^ Recently, stem cell transplantation has been a focus in the treatment of IS. Stem cells possess self‐renewal and differentiation potential. Besides, they secrete beneficial cytokines that stimulate endogenous repair post IS.^5^ However, the efficacy of stem cell therapy is constrained by low cell survival, uncontrolled differentiation, and ineffective integration into host tissues. Despite optimization of transplanted stem cells—including cell type selection, dosage, delivery route, timing, and even genetic engineering—these aforementioned limitations remain inadequately resolved.

The growth of tissue engineering has introduced increasing applications of biomaterials into stroke treatment, while creating novel perspectives for stem cell transplantation. The post‐stroke formation of ischemic cavities naturally accommodates local delivery of both stem cells and biomaterials, effectively circumventing the low targeting efficiency associated with systemic administration.^7^ Structure–activity relationships (SARs) encompass a suite of advanced drug design methodologies that analyze how a molecule's chemical structure influences its biological activity.^8^ We invoke this concept to demonstrate how precisely engineered biomaterial properties—including physical (e.g., porosity, stiffness, topology), chemical (e.g., pharmaceutical agents, crosslinkers), and biological (e.g., peptides, bio factors) characteristics—can synergistically enhance the neurorestorative potential of transplanted stem cells. Unfortunately, few biomaterials specifically for stroke have yet been approved for clinical trials (clinicaltrials.gov).

This review first systematically elaborates on the pathological changes during the three phases following IS. Subsequently, we identify potential therapeutic targets within each phase, thereby providing a conceptual framework for designing biomaterials that synergize with endogenous repair mechanisms. Second, from the stem cell perspective, we focus on: (1) stem cell types, (2) delivery strategies, (3) therapeutic mechanisms, and (4) factors limiting treatment efficacy. This systematic analysis forms the foundation for biomaterial integration. Next, we provide a comprehensive overview of animal studies employing biomaterial‐assisted stem cell therapy for stroke in recent years, with particular focus on the diverse functional roles of biomaterials. Then, we list current relevant clinical trials in detail. While the scarcity of clinical studies highlights the multifaceted challenges in clinical translation, we must equally recognize the considerable therapeutic potential this approach holds.

## 2 PATHOLOGIC CHANGES AND REPAIR MECHANISMS OF ISCHEMIC STROKE

**FIGURE 1:**
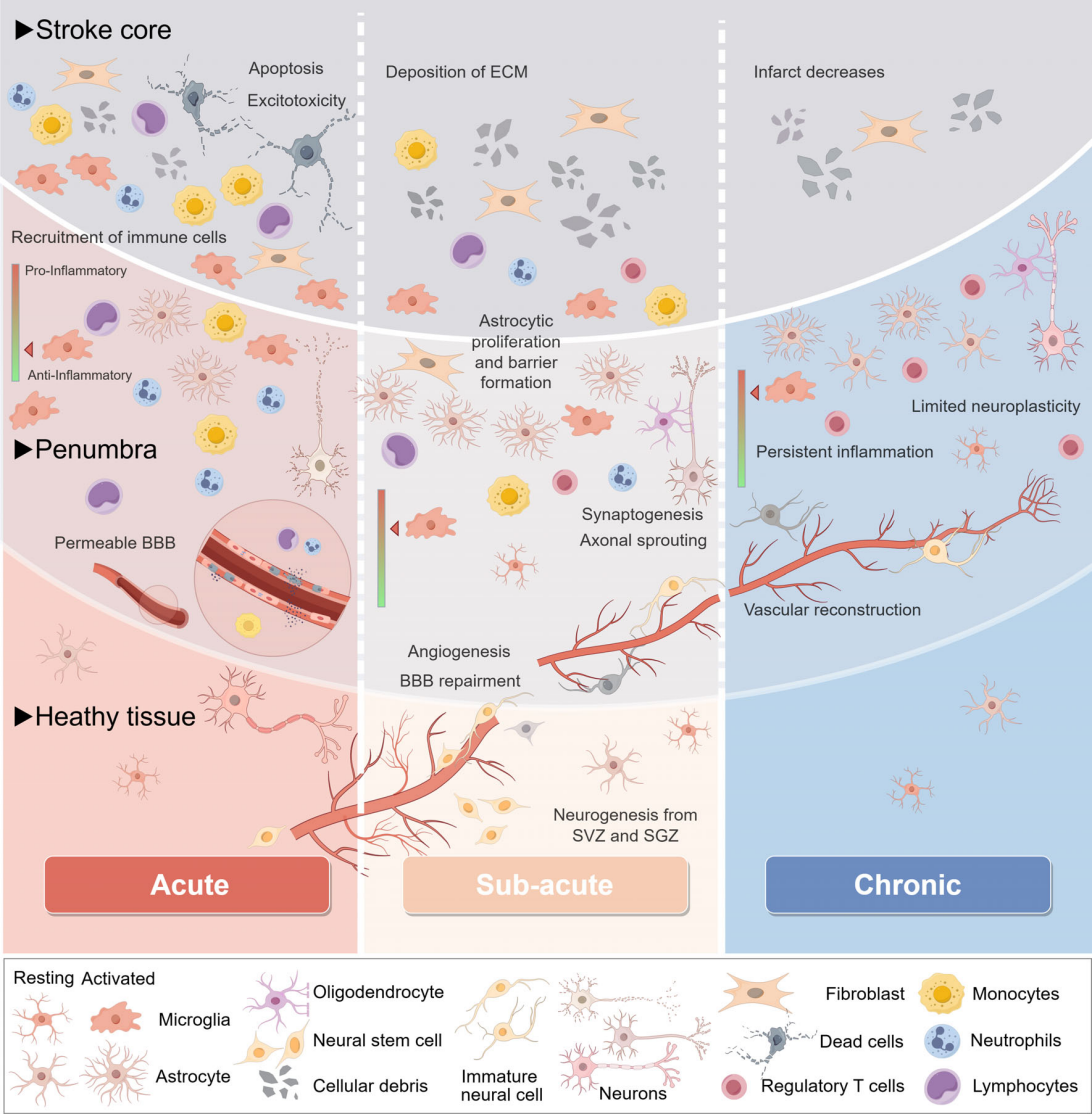
The pathological changes for ischemic stroke. Within minutes of ischemic stroke onset, cell death begins. During the acute phase, microglia respond rapidly, and peripheral immune cells infiltrate the brain through the compromised blood–brain barrier. In the subacute phase, inflammation gradually diminishes, and a glial scar, primarily composed of reactive astrocytes, microglia, and extracellular matrix, begins to form. A cavity slowly develops at the infarct site. Vascular and neural regeneration processes become active, with synaptic plasticity and axonal sprouting being promoted. Neural stem cells in the subventricular zone (SVZ) and dentate gyrus (SGZ) proliferate and migrate to the peri‐infarct site. In the chronic phase, repair of vascular and neural networks is limited, although Treg cells, which infiltrated during the subacute phase, increase in number and contribute to neural repair.

Following ischemic stroke, loss of blood flow leads to a deficiency in oxygen and nutrients. Neuronal death occurs within minutes and a series of damage and repair processes starts. This can be defined in three stages: acute, subacute, and chronic^7^ (Figure 1). Effective treatments must be designed to address this dynamic and complex pathological process.

### 2.1 Acute phase: Challenging and destabilizing environment

During the acute phase (within 1 week of human IS onset^6^, ^7^), hypoxic conditions lead to increased anaerobic metabolism, ionic dysregulation, and abnormal neurotransmitter release.^9^ Excessive extracellular glutamate activates severe cell injury and apoptotic pathways, known as excitotoxicity.^10^ Oxidative stress and DNA damage further mediate this process.^9^ A large number of injured or necrotic neurons release molecules (including reactive oxygen species(ROS)) that activate endothelial cells, leading to disruption of the blood–brain barrier (BBB).^11^ These molecules also trigger the activation of microglia first and peripheral immune cells (neutrophils, macrophages, lymphocytes) that secrete abundant pro‐inflammatory cytokines (such as tumor necrosis factor alpha [TNF‐α], interleukin‐1 beta [IL‐1β], matrix metalloproteinase‐9 [MMP9]) and infiltrate brain tissue^12^ (Figure 1). These endogenous inflammatory molecules are called damage‐associated molecular patterns (DAMPs),^13^ initiate the inflammatory response, and peak within a week. The intense inflammatory reaction can lead to systemic immunosuppression and complications such as stroke‐associated pneumonia.^14^ In summary, during the initial minutes to hours following IS, the infarct core experiences: (1) precipitous decline in cerebral blood flow (CBF) and tissue oxygen tension (PO₂),^15^, ^16^ (2) massive accumulation of ROS, and (3) exponential increase in pro‐inflammatory cytokines. Due to the rapid changes and harmful environment, the acute phase is not suitable for biomaterial or stem cell transplantation.

### 2.2 Subacute phase: Key window for endogenous repair and exogenous intervention

During the subacute phase (within 3 months of human IS onset^7^), inflammation gradually subsides. Microglia, key immune cells after IS, can transition between pro‐inflammatory (secreting pro‐inflammatory cytokines, ROS, MMPs) and anti‐inflammatory (secreting transforming growth factor beta [TGF‐β], interleukin‐4 [IL‐4], interleukin‐10 [IL‐10], and other cytokines or nutritional factors) phenotypes.^17^ Microglia engage in close bidirectional communication with neurons,^18^, ^19^ astrocytes,^20^ oligodendrocytes,^21^ and endothelial cells.^22^, ^23^ While astrocytes induce peripheral inflammatory cell infiltration during the acute phase, they contribute to neuroprotection and repair during the recovery phase by releasing antioxidants, neurotrophic factors, and functional mitochondria.^24^, ^25^ Thus, modulating glial cells towards a reparative phenotype has a widespread impact on the brain's internal environment. Regulatory T cells (Tregs) begin to significantly infiltrate the damaged brain in the subacute phase and continue to increase for months post‐IS, with Tregs playing a crucial role in immune suppression.^26^

During the subacute phase, the activity of angiogenesis around the infarct increases and persists for several weeks. This newly formed vascular network serves as a pathway for immature neurons to reach the damaged area.^27^ Studies show that after IS, endogenous neurogenesis occurs in the subventricular zone (SVZ) and the dentate gyrus (DG).^28^, ^29^ However, the number of neural stem cells (NSCs) that successfully migrate to and mature in the penumbra region is very limited.^30^ Thus, biomaterials targeting vascular repair can both restore blood flow in the ischemic penumbra and support the migration of NSCs.^31^ During this phase, glial scar gradually forms, preventing further expansion of the infarct core.^32^ The infarct core eventually creates a cavity, providing a suitable site for in situ implantation of biomaterials and stem cells.^7^ While synaptic and axonal plasticity are enhanced during this period, glial scar tissue hinders the extension of axonal sprouts into the infarct zone. It releases factors that inhibit axonal growth and cell infiltration,^33^ which also adversely affects the integration of in situ transplanted stem cells.

The aforementioned endogenous repair mechanisms often fall short of achieving satisfactory functional recovery and are not long‐lasting. Thus, biomaterials should act as scavengers or amplifiers to isolate harmful signaling molecules, enhance repair effects, and extend the beneficial period to support and promote the neural repair process.^34^ For instance, there is an ROS‐triggered hyaluronic acid and platelet lysates (Pls) composite hydrogel encapsulating matrix MMPs‐responsive triglycerol monostearate nanoparticles loaded with docosahexaenoic acid.^35^ In this scaffold, (1) the borate ester bonded hydrogel could respond to ROS and relieve oxidative stress; (2) TD nanoparticles could be enzymatically cleaved by overexpressed MMPs; (3) DHA can not only inhibit neuroinflammatory but also take part in promoting neurogenesis; (4) Pls gel contains an abundance of growth factors that target revascularization. The hydrogel was demonstrated to have promising therapeutic effects on remodeling the pathological environment by transforming the hostile state into a pro‐regenerative one in the infarct site in IS mouse.

### 2.3 Chronic phase: A plateau with limited interventions

In the chronic phase, 3 months after stroke onset, Tregs play a crucial role in maintaining white matter integrity and suppressing excessive astrocyte proliferation.^36^ The BBB gradually rebuilds. However, repair activities significantly weaken, leaving only limited neuroplasticity^7^ (Figure 1). While clinical rehabilitation strategies, based on motor relearning and neuroplasticity enhancement,^37^ can partially improve motor function in IS patients, they often fall short of meeting the rehabilitation needs of most patients. Therefore, there is an urgent need for effective reparative interventions following IS.

## 3 TRANSPLANTED STEM CELL THERAPY FOR ISCHEMIC STROKE

### 3.1 Types of stem cells

**FIGURE 2:**
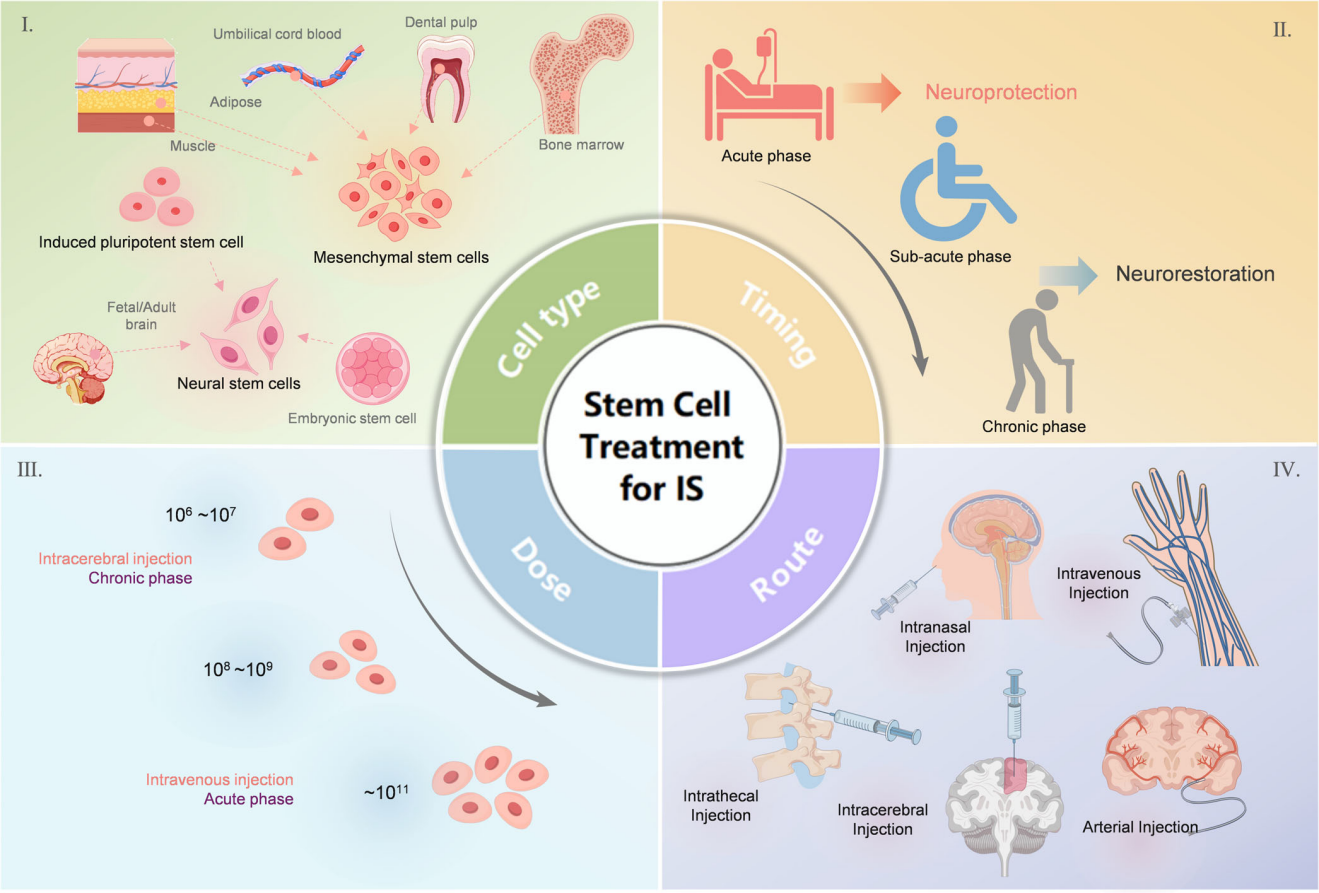
Advances in stem cell treatment for ischemic stroke in clinical trials. (I) For stroke transplantation, cells are primarily categorized into three types: mesenchymal stem cells, neural stem cells, and induced pluripotent stem cells. (II) Stem cell transplantation can occur during the acute phase of stroke (primarily neuroprotective effects), subacute phase, and chronic phase (primarily for neuroplasticity). (III) The number of transplanted cells ranges from 10^6^ to 10^9^, influenced mainly by the timing and method of transplantation. (IV) Transplantation routes include intravenous, intra‐arterial, intrathecal, intracerebral, and intranasal injections, with intravenous and intra‐arterial being the most commonly used.

Currently, stem cells used for IS treatment can be broadly categorized into three types^32^: mesenchymal stem cells (MSCs), neural stem cells (NSCs), and induced pluripotent stem cells (iPSCs) (Figure 2).

**FIGURE 3:**
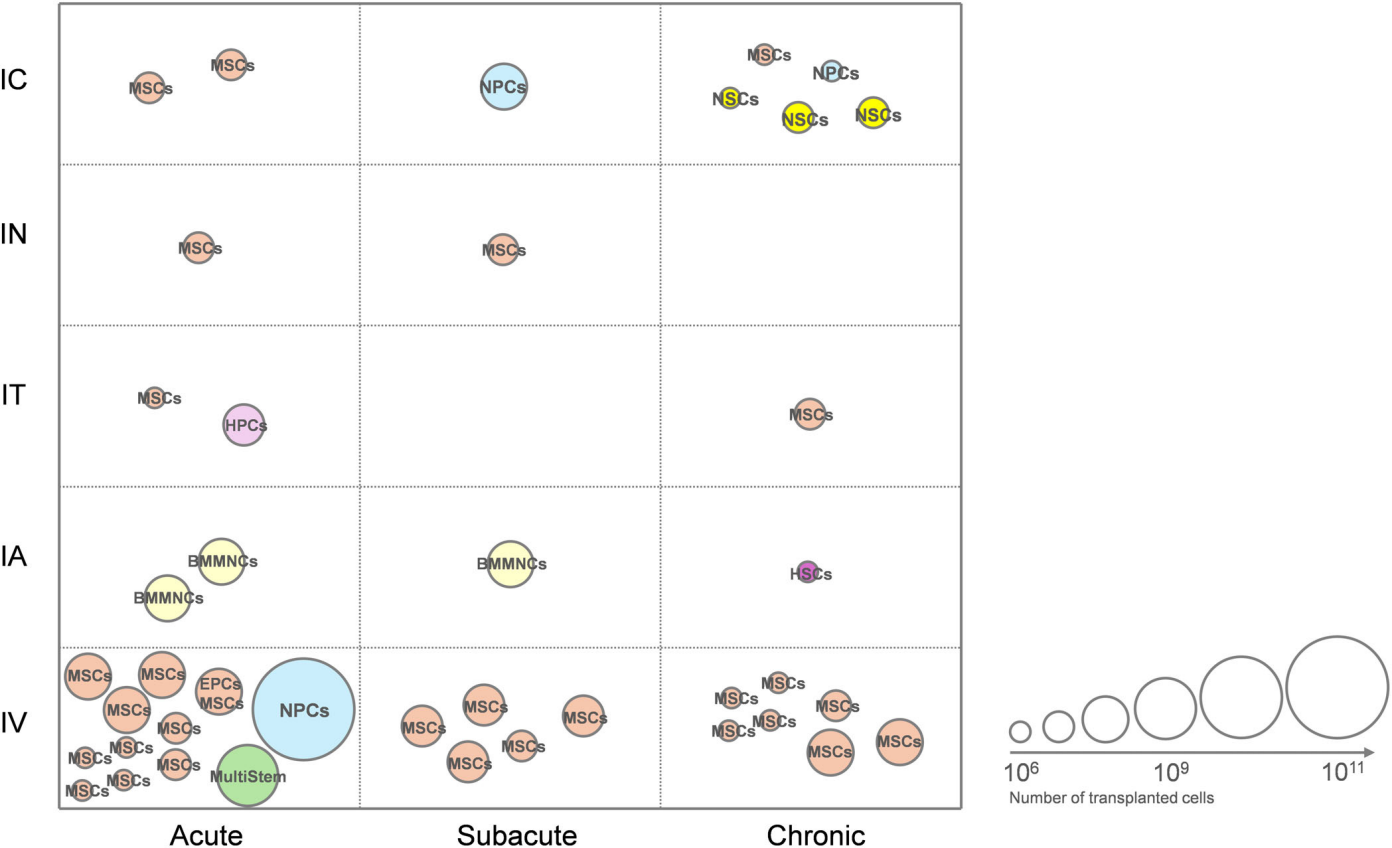
Delivery strategies of stem cells for ischemic stroke in clinical trials. Acute phase‐IC: NCT02767817, NCT05008588; Acute phase‐IN: NCT03356821; Acute phase‐IT: NCT05292625, NCT03735277; Acute phase‐IA: NCT00473057, NCT00761982; Acute phase‐IV: NCT03545607, NCT04093336, NCT01678534, NCT04811651, NCT00875654, NCT05850208, NCT04280003, NCT02605707, NCT05697718, NCT05008588, NCT06138210, NCT01468064, NCT00875654; Subacute phase‐IC: NCT02117635; Subacute phase‐IN: NCT03356821; Subacute phase‐IA: NCT00473057; Subacute phase‐IV: NCT01461720, NCT00875654, NCT06129175, NCT05292625, NCT04811651; Chronic phase‐IC: NCT01714167, NCT03296618, NCT03629275, NCT06299033, NCT04631406; Chronic phase‐IT: NCT05292625; Chronic phase‐IA: NCT01273337; Chronic phase‐IV: NCT04953663, NCT02580019, NCT04590118, NCT05850208, NCT01297413, NCT04811651. The figure exclusively displays clinical trials with quantifiable transplanted cell numbers in the available records.

MSCs can be extracted from peripheral tissues such as bone marrow, adipose tissue, muscle, dental pulp, and umbilical cord blood.^38^ They are minimally immunogenic and pose fewer ethical concerns, making them widely used in animal models of IS.^39^ MSCs and their extracellular vesicles can regulate the inflammatory environment,^40^ reduce neuronal death,^41^ and modulate neural and vascular remodeling^42^, ^43^, ^44^ after cerebral ischemia. NSCs can be derived from adult or fetal brain tissue, as well as from embryonic and iPSCs.^45^ NSCs are adapted to the neural environment and maintain a stable neuronal differentiation profile, primarily functioning through cell replacement.^46^, ^47^ iPSCs have unlimited self‐renewal and pluripotency but carry a risk of tumor formation after transplantation.^48^ iPSC‐derived neural epithelial‐like stem cells can differentiate into cortical neurons and form functional synapses with host cells,^49^ but most studies suggest iPSC transplantation mainly has a “bystander” effect.^37^ Multilineage differentiating stress‐enduring (Muse) cells^50^ are adult stem cells first reported in 2010. These cells are a subpopulation of MSCs that are positive for both pluripotent and mesenchymal markers. Notably, Muse cells are pluripotent while considered nontumorigenic.^50^ Preclinical studies have shown that Muse cells are able to migrate to the peri‐infarct and survive for a longer period of time, have better neural differentiation, and integrate into the motor sensory cortex compared to the non‐MUSE cells.^51^, ^52^, ^53^ Furthermore, additional stem cell products have been explored in clinical trials, including peripheral hematopoietic stem cell (HSCs, NCT01518231), bone marrow mononuclear cells (BMMNCs, NCT00761982, NCT00473057, NCT02425670), MultiStem (an adult stem cell investigational product, NCT03545607) (Figure 3). Stem cell reprogramming enhances the efficacy of stem cell transplantation by secreting beneficial cytokines, such as brain‐derived neurotrophic factor (BDNF),^54^ glial cell line‐derived neurotrophic factor (GDNF),^55^, ^56^ vascular endothelial growth factor (VEGF),^57^ hepatocyte growth factor (HGF),^58^ placental growth factor (PIGF),^59^ and angiopoietin‐1 (ANG‐1),^60^ or contribute to reducing the risk of tumor formation^61^ and regulating differentiation.^62^, ^63^, ^64^

In summary, current cell‐based stroke therapeutics use diverse cellular sources, requiring careful consideration of: (1) harvesting potential, (2) proliferative capacity, (3) tissue compatibility, and (4) therapeutic mechanisms when selecting optimal candidates. To enhance or diversify stem cell functionality, engineered cell products are being increasingly employed. However, their clinical translation faces new challenges regarding production costs and batch‐to‐batch variability.

### 3.2 Delivery strategies

Beyond cell type, stroke stem cell therapy must address transplantation route, cell dosage, and timing (Figure 2), as these factors influence each other. Despite increasing clinical research, identifying the optimal combination of cell type, dose, timing, and route remains difficult.

Currently, there are five primary routes for stem cell transplantation in stroke: intravenous (IV), intra‐arterial (IA), intracerebral (IC), intrathecal (IT), and intranasal (IN)^3^ (Figures 2 and 3). Generally, IV is the most commonly used due to its minimal invasiveness and simplicity. Though IA can deliver more cells to the lesion, it may cause vascular blockage by cell clumps. Both IV and IA are limited by the pulmonary first‐pass effect and the selective permeability of the BBB, resulting in few cells reaching the brain.^65^ IT allows cells to permeate the brain parenchyma from the cerebrospinal fluid. However, IT may lead to complications such as hydrocephalus and cerebrospinal fluid leakage.^66^ IC offers the highest delivery efficiency but can cause additional brain damage, leading to motor impairments and seizures, and is less commonly used in clinical trials.^67^, ^68^ In animal studies, stem cell delivery methods appear more aggressive. Clinical (NCT03356821, ChiCTR1900027199, ChiCTR1900022741) and preclinical research on IN stem cell grafts are limited and may require hyaluronidase to enhance nasal mucosa permeability.^69^, ^70^, ^71^

Most clinical trials perform cell transplantation in the acute or subacute phase, with 7‐day post‐IS being considered optimal for cell delivery in pre‐clinical trial.^45^ However, optimal timing may vary with different animal model.^72^ Transplantation methods are influenced by timing; for example, most clinical trials using IC are conducted during the chronic phase.^73^ Clinical trials use stem cell doses ranging from 10^6^ to 10^11^, with animal models generally using fewer cells due to smaller brain and infarct volumes.^74^ IV or IA methods typically administer more cells than IC (Figures 2 and 3).

### 3.3 Therapeutic mechanisms

**FIGURE 4:**
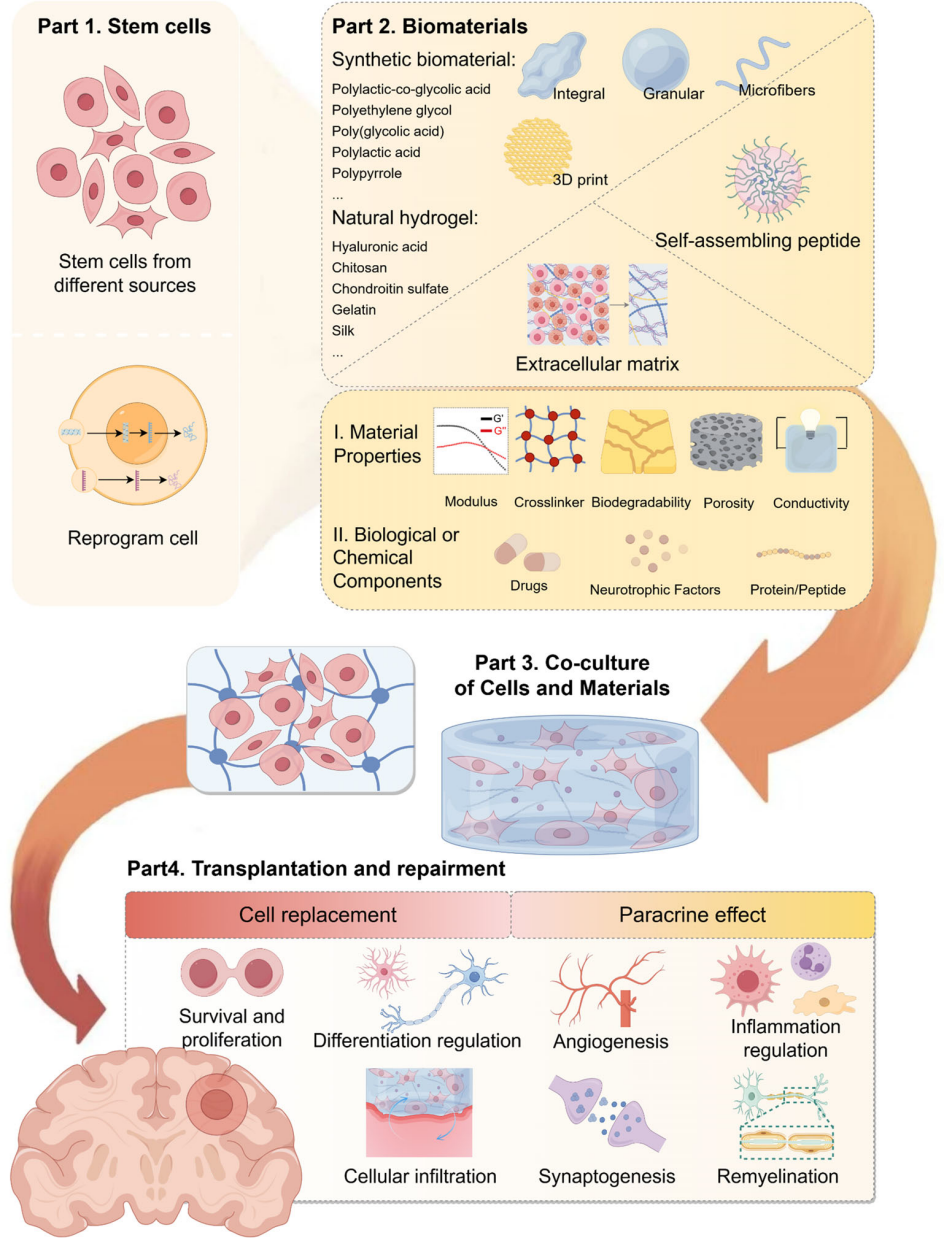
Biomaterials as cell carriers for ischemic stroke repair. A variety of biomaterials can serve as carriers for stem cell transplantation, either by surface loading or encapsulating the cells. Modifying the design parameters of these biomaterials—such as their physical properties and biological or chemical components—can create a more suitable external environment for the cells. The combined use of biomaterials and cells significantly enhances the survival and migration of the transplanted cells compared to cell‐only approaches. Additionally, the synergistic effects of biomaterials and cells promote various aspects of neurorepair.

From a mechanistic perspective, transplanted stem cells mediate tissue repair through two fundamental pathways: cell replacement and paracrine effect (Figure 4).

Cell replacement involves transplanting stem cells that differentiate into functional cells to compensate for lost brain tissue, including both neural cells and vascular endothelial cells. For instance, in rat permanent middle cerebral artery occlusion (MCAO) models, transplanted bone marrow‐derived mononuclear cells (MNCs) demonstrate differentiation capacity into smooth muscle cells (SMCs) and endothelial cells (ECs), indicating their participation in angiogenesis.^75^ Implanting triple‐layer adipose‐derived MSCs sheets onto the surface of the infarcted brain region can differentiate into pericytes, promoting vascular reconstruction in the peri‐infarct area of rats.^76^ Transplanted NSCs can mature within the brain and form synaptic connections with neighboring neurons, enhancing nerve impulse conduction.^77^ Biomaterials can significantly potentiate this therapeutic process.^9^ Myelination is the process of covering neuronal axons with a myelin sheath and is carried out by oligodendrocytes (OLs). This lipid‐rich insulation significantly enhances axonal conduction velocity.^78^ Less focus is given to the differentiation of transplanted NSCs into OLs compared to neurons and astrocytes. However, cell damage following IS inevitably leads to demyelination and remyelination is also essential. Transplantation of exogenous oligodendrocyte progenitor cells (OPCs) derived from iPSCs can form functional myelin sheaths in animal brains, aiding in remyelination and recovery of neurological function in MCAO mice.^79^, ^80^

Some studies have found that functional recovery after brain ischemia is not directly related to the long‐term survival of transplanted stem cells,^81^ suggesting that stem cells also exert other therapeutic effects—“Paracrine effect.” Stem cells secrete cytokines, chemokines, and exosomes, which provide anti‐apoptotic, anti‐inflammatory, and immunomodulatory effects, and mobilize neurogenesis.^82^ Similarly, biomaterials can strategically intervene in these processes. Intravenous administration of neural progenitor cells (NPC)‐derived extracellular vesicles in MCAO mice demonstrated significant anti‐inflammatory effects through suppression of the MAPK signaling pathway. However, the therapeutic efficacy of exosomes requires modification with infarct‐targeting peptides to improve their bioavailability.^83^ Intracerebral local delivery of curative factors via biomaterial systems significantly reduces systemic circulation losses. For instance, exosomes from MSCs loaded into Gelatin methacryloyl hydrogel microneedles effectively reduce local neuroinflammation, promote angiogenesis, and decrease glial scar formation in MCAO rats.^84^ Erythropoietin delivered from hyaluronan/methyl cellulose post‐stroke resulted in attenuated inflammatory response, reduced stroke cavity size, and promoted neurogenesis.^85^

While our discussion above focuses on the tissue‐repair mechanisms of stem cell transplantation, these very mechanisms also represent prime targets for biomaterial‐assisted therapy. The critical challenge lies in strategically leveraging the synergistic interaction between stem cells and bioactive scaffolds.

### 3.4 Factors affecting therapeutic efficacy

Despite the advantages and disadvantages of various stem cell transplantation methods, they all face common issues such as low cell survival, inadequate migration to the lesion site, and uncontrollable differentiation.^73^ For example, bone marrow mononuclear cells transplanted via the IA route are undetectable in the brain within 24 h.^86^ Additionally, the therapeutic efficacy of stem cell transplantation largely depends on their widespread distribution within the host brain.^87^ The glial scar that gradually forms after ischemic stroke hinders the infiltration of transplanted stem cells into the host tissue.^7^ Nutrient delivery also suffers from uncontrollable release issues. Delivering biological factors requires biomaterial interventions to ensure sustained release and efficacy.^88^, ^89^ Overall, these limitations can be significantly improved through the use of biomaterials.

## 4 BIOMATERIALS IN STEM CELL THERAPY FOR ISCHEMIC STROKE

### 4.1 Carriers of transplanted stem cell

Tissue engineering employs hydrogels, microporous scaffolds, and nanofibers made from proteins, peptides, polysaccharides, and polymers as functional carriers for stem cell transplantation (Figure 4). These bioengineering scaffolds are infusing new vitality into stem cell transplantation research.^4^

Hydrogels are widely used biomaterials composed of cross‐linked polymeric networks with high water content. They support cell adhesion and allow for the simple diffusion of biological factors.^90^ Hydrogels used as cell carriers can be classified into two types: natural (e.g., hyaluronic acid, collagen, fibrin, gelatin, and alginate) and synthetic (e.g., polyethylene glycol).^89^ By modifying the material composition, polymerization parameters, macroscopic shape, and microscopic structure, hydrogels can be designed to mimic the structural features of brain tissue. However, natural hydrogels may lack mechanical strength and degrade rapidly in vivo,^91^ though some degradation products can also exert biological effects.^92^ Hydrogel microfibers can guide cell growth and are easier to inject.^93^, ^94^, ^95^ Additionally, 3D printing enables the creation of hydrogels with more complex geometries.^96^, ^97^ Hydrogels can be shaped into microgel particles with micron‐sized pores that align with cell dimensions, enhancing cell infiltration and tissue regeneration.^98^, ^99^, ^100^ Anisotropic rod‐shaped microgels with different pore shapes and better interconnectivity can improve cell and vessel invasion compared to spherical particles.^99^ Based on granular hydrogel, microporous annealed particle (MAP) provides a soft environment with higher specific surface and adsorption capacity, contrasting with bulk hydrogels or rigid scaffolds.^98^, ^101^, ^102^ Studies have shown that MAP scaffolds are porous and recruit neural progenitor cells to the stroke cavity after injection into the stroke core.^102^

While hydrogels can mimic the mechanical properties of brain tissue, they lack key proteins found in the natural extracellular matrix (ECM). ECM‐based biological scaffolds derived from decellularized tissues offer natural advantages for neural repair.^103^ When ECM scaffolds are gelled in situ at the infarct site in MCAO rats, they rapidly induce cell infiltration^104^, ^105^ and significantly reduce infarct volume.^104^ ECM can also serve as a carrier for stem cells or other material particles,^73^ but fewer studies for IS are available.

Self‐assembling peptides (SAPs) spontaneously form stable nanofiber gels in aqueous solutions. SAP scaffolds based on the laminin‐derived IKVAV peptide have mechanical properties compatible with brain tissue, supporting the survival and differentiation of NSCs into functional neurons. This results in reduced brain atrophy and promotes long‐term recovery of motor functions in stroke animals.^107^ RADA(16)‐IKVAV can self‐assemble into a nanofibrous morphology with a bilayer β‐sheet structure and direct the encapsulated neural stem cells (NSCs) adhesion and then toward neuronal differentiation.^106^

Polylactic‐co‐glycolic acid (PLGA) is an FDA‐approved polymer with excellent biocompatibility. Upon exposure to the physical environment, PLGA will be hydrolyzed into lactic and glycolic acids, which are naturally occurring metabolites. However, unmodified PLGA exhibits hydrophobicity and poor cell adhesion due to hydrophobicity and negative surface charge; surface modification or incorporation with other materials is necessary.^108^, ^109^ When combined with hydrophilic polyethylene glycol (PEG), PLGA forms PLGA‐PEG micelles. PEG modification of PLGA enables self‐assembly into micellar nanoparticles with hydrophilic shells and hydrophobic cores, where better cell adhesion occurs. Loading these micelles with NSCs for in situ transplantation in IS mouse has been shown to reduce infarct volume and promote neurological recovery.^110^ Additionally, synthetic materials such as polylactic acid,^98^ poly(glycolic acid),^111^ and polypyrrole^112^ are also utilized as cell carriers in IS treatment.

### 4.2 The role of biomaterials in stem cell therapy

#### 4.2.1 Enhancing graft cell retention, survival, and proliferation

**FIGURE 5:**
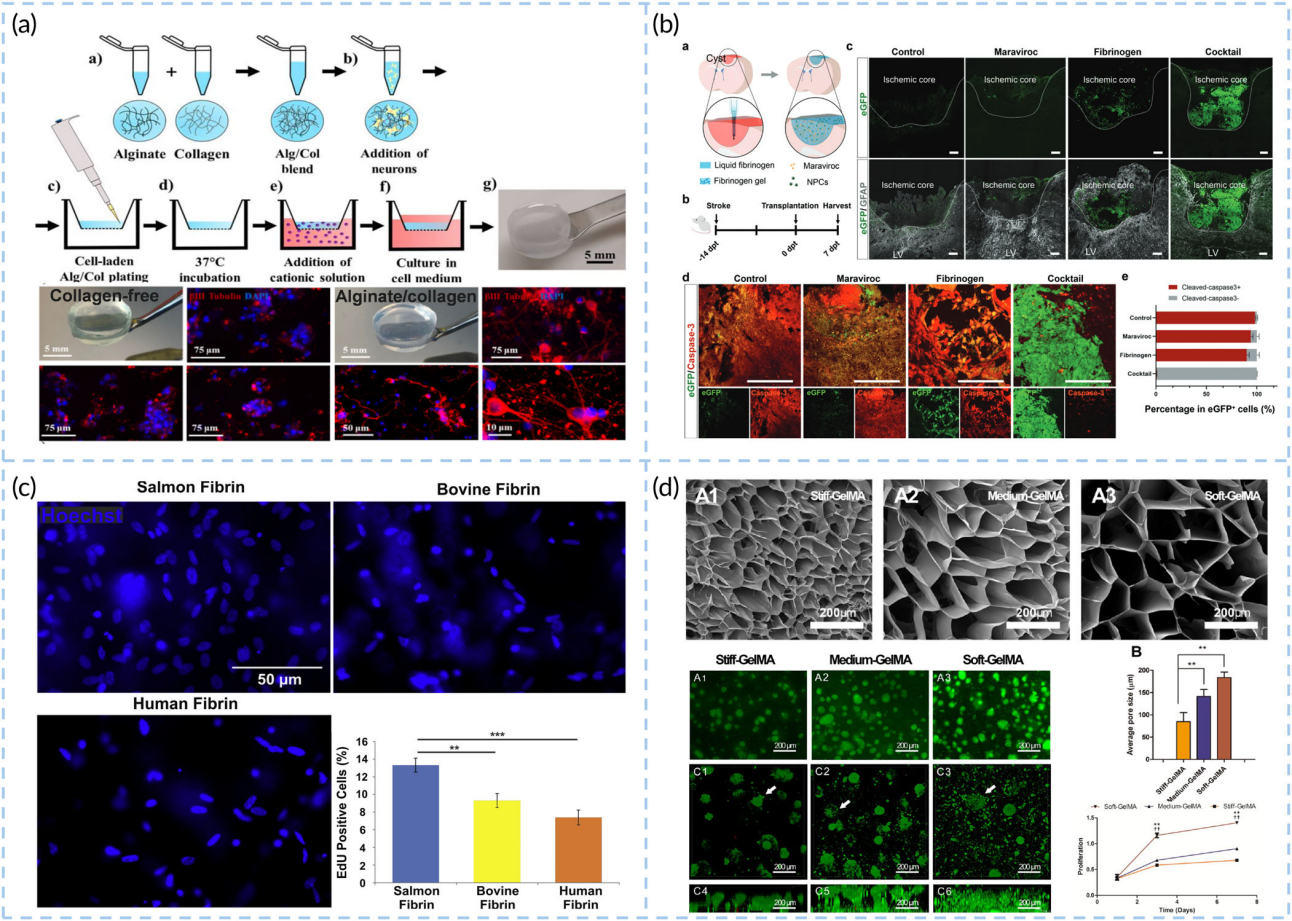
Biomaterials enhance graft cell adhesion, retention, and proliferation. (a) Alginate/collagen hydrogels support better adhesion of iPSC‐derived neurons than collagen free hydrogel. Reproduced with permission from Reference 113. (b) A chemical cocktail consisting of fibrinogen and maraviroc promote the survival of the transplanted NPCs in the ischemic core of the mouse cerebral cortex. Reproduced with permission from Reference 118. (c) Proliferation was significantly greater for human neural stem/progenitor cells in salmon fibrin than cells in mammalian. Reproduced with permission from Reference 119. (d) Proliferation of neural stem cells in the GelMA hydrogels was found significantly increased as the modulus of the hydrogels decreased. Reproduced with permission from Reference 123.

Due to the detrimental microenvironment within brain infarct cavities, maintaining cell retention, survival, and proliferation presents significant challenges for cell carriers. To address these issues, cell scaffolds must first exhibit superior cell‐loading capacity. Gelatin and collagen hydrogels contain integrin binding sites for cell‐matrix interactions to promote cell adhesion and proliferation^113^ (Figure 5a). Incorporating collagen fibrils into an alginate hydrogel (lacking cell‐adhesive sequences) by physical mixing successfully supported attachment of human iPSC‐derived neurons.^114^ Some natural hydrogels offer abundant cell adhesion sites, while synthetic materials can incorporate RGD (Arg‐Gly‐Asp) adhesion peptides or be coated with adhesion proteins.^115^, ^116^ Biomaterials may enhance cell retention through integrin‐mediated mechanisms. A polylactic acid polymeric rough microfibrous delivery increased MSCs retention at the injury site. The scaffold upregulated α6‐integrin and CXCL12 production, which may be the cause for greater cell retention at the lesion site.^98^

Scaffolds coated with laminin have been shown to improve stem cell survival both in vitro and in vivo.^116^ Optimizing the concentration of adhesion peptides derived from laminin (IKVAV and YIGSR) and fibronectin (RGD) can enhance the survival and infiltration of iPSC‐NPCs, and even promote their differentiation into neurons.^117^ Additionally, fibrinogen gels loaded with Maraviroc can protect transplanted NPCs from apoptosis induced by pro‐inflammatory factors by inhibiting the CCR5 pathway^118^ (Figure 5b).

Scaffold parameters (chemistry/physics) differentially regulate proliferation. Proliferation was significantly greater for human NPCs in salmon fibrin than for cells in mammalian fibrin^119^ (Figure 5c). Electroconductive materials also influence cellular proliferation. Polypyrrole (PPy) conductive polymers loaded with pre‐electrostimulated human NPCs delivered to infarct sites can promote functional recovery in rats. Ingenuity pathway analysis (IPA) showed the most significant pathways changed by electrical stimulation included those with effects on cell cycle/proliferation, survival.^112^ Biomaterials loaded with growth factors or drugs further support cell proliferation.^34^, ^120^ For instance, seeding NSCs onto poly(trimethylene carbonate)_15_‐F127‐poly(trimethylene carbonate)_15_ (PTMC_15_‐F127‐PTMC_15_, PFP) thermosensitive hydrogel scaffolds loaded with BDNF, nerve growth factor (NGF), and neurotrophin‐3 (NT‐3) results in sustained release of these factors for over 14 days in the brain, thereby enhancing the therapeutic effects of NSCs.^121^ A GelMA‐T hydrogel (developed by introducing taurine on gelatin methacryloyl [GelMA]) plays a positive role in promoting NSC proliferation.^122^ Moreover, soft GelMA hydrogels with larger surface pore sizes enhance higher neural stem cell (NSC) proliferation rates than stiff hydrogels^123^ (Figure 5d).

#### 4.2.2 Regulating the differentiation fate of stem cells

**FIGURE 6:**
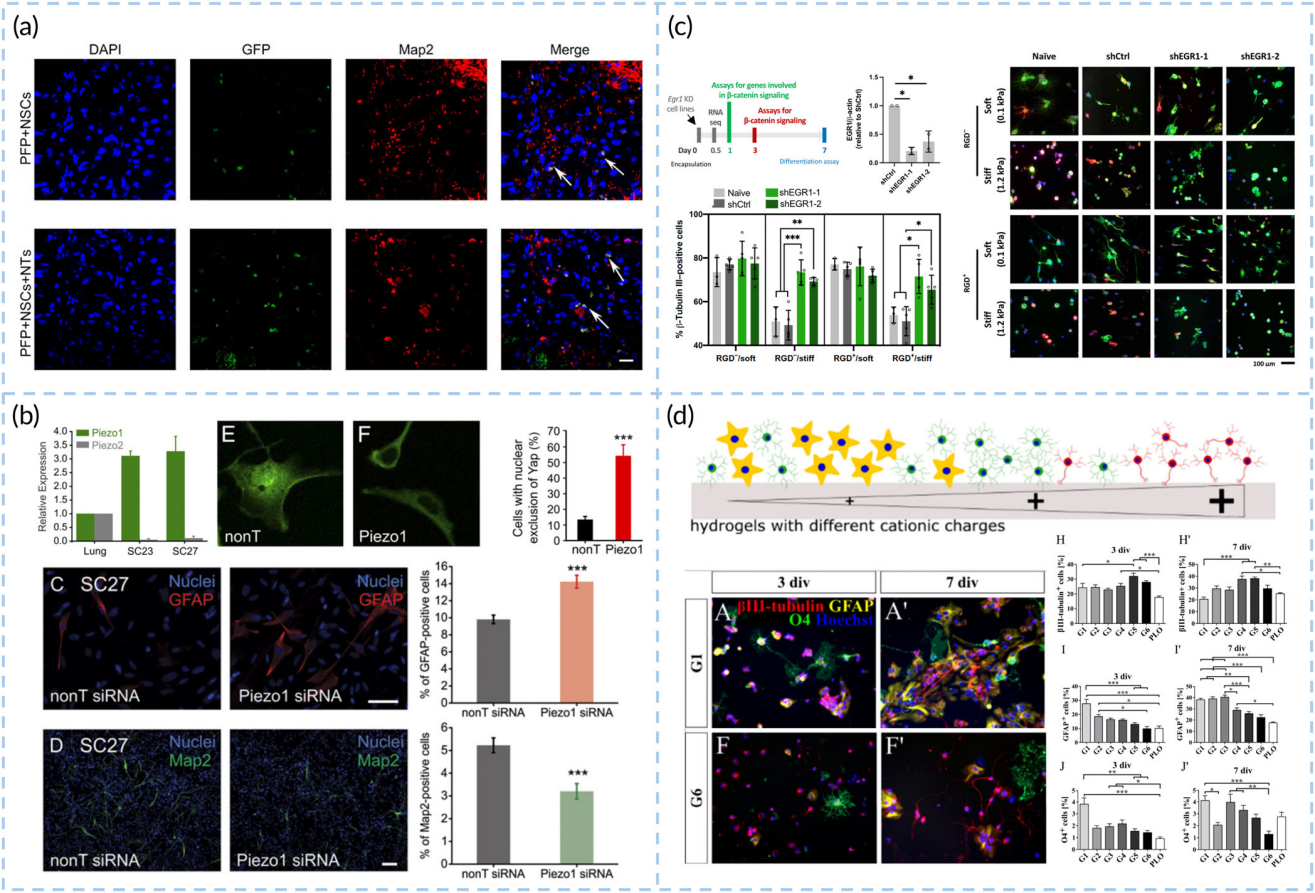
Biomaterials regulate differentiation fate of stem cells. (a) PFP polymer hydrogels loaded with neurotrophic BDNF, NGF, NT3 induced the differentiation of grafted NSCs into mature neurons. Reproduced with permission from Reference 121. (b) Piezo1 is expressed by brain‐derived NPCs and directs NPCs lineage commitment by modulating YAP nuclear localization. Reproduced with permission from Reference 128. (c) Stiffness of ECMs regulate NSCs differentiation by driving β‐catenin nuclear localization and activity in 3D hydrogel. Reproduced with permission from Reference 133. (d) A synthetic hydrogel system with a cationic group concentration ranging from 0.06 to 0.91 μmol/mg was prepared. The adhesion of neural cells was promoted by a higher concentration of cationic molecule hydrogel. Reproduced with permission from Reference 134.

Directly compensating for damaged neural circuits after stroke through transplanted stem cells is a key objective of numerous studies. The regulation of neural stem cell fate is a meticulously controlled process, influenced by extracellular biological and physical signals^111^ (Figure 6).

Biological factors such as BDNF, fibroblast growth factor (FGF), stromal‐derived factor 1 (SDF‐1), transforming growth factor β (TGFβ), and Wnt/Nodal inhibitors like Dickkopf‐1 and Lefty‐1, as well as microRNAs such as miR‐125 and miR‐9, all serve as regulators of neural differentiation.^113^, ^124^, ^125^, ^126^ For example, the transplantation of bFGF‐loaded chitosan gels into the infarct cavity of Photochemical (PT) stroke rats promotes the differentiation of endogenous NSCs into mature neurons around the infarct area.^88^ PFP polymer hydrogels loaded with BDNF, NGF, NT3, and NSCs could sustain the release of growth factors, which induced the differentiation of NSCs into mature neurons^121^ (Figure 6a). The impact of ECM mechanical forces on cell behavior is undeniable.^126^ Typical mechanosensitive receptors like integrins and cadherins,^127^ as well as mechanosensitive ion channels such as the Piezo family,^129^ transduce mechanical stimuli into biochemical signals. These signals activate intracellular response pathways that influence cell behavior.^130^, ^134^ Elastic modulus has a major impact on stem cell lineage choice.^122^ When MSCs grow on polyacrylamide gels with a gradient of stiffness from low to high, the cytoskeleton rearranges, and the cells tend to differentiate into neural, muscular, and osteogenic lineages, respectively.^131^ On low‐modulus substrates, Piezo1‐dependent Ca^2+^ influx increases, leading to greater neuronal differentiation and less astrocytic differentiation in NPCs^128^ (Figure 6b). Topographical cues of biomaterials can induce cytoskeletal rearrangement, potentially mediated by integrins and the Rho signaling pathway, which affects YAP nuclear localization.^131^, ^132^ Current research reveals that, under 3D culture conditions, stem cells perceive ECM signals differently compared to 2D environments.^126^ Perhaps by directly sensing the surrounding mechanical forces independent of integrins, cells can induce cytoskeletal rearrangement and activate signaling pathways, such as Wnt/β‐catenin, that regulate neural differentiation^133^ (Figure 6c). Additionally, the electrical conductivity of materials (a synthetic hydrogel system based on 4‐acryloylmorpholine [AMor], N,N′‐bis(methacryloyl)cystamide [BMAC], and trimethylaminoethyl acrylate [TMAEA]) can influence differentiation. NPCs seeded onto gels with varying concentrations of cationic groups show increased differentiation into neurons on high‐cation gels^134^ (Figure 6d).

It is important to note that after a stroke, not only neurons are lost, but also endothelial cells and various types of glial cells. Therefore, strictly limiting the differentiation fate of transplanted cells does not align with the principles of repair.^117^ Typically, there is a disconnect between in vitro studies on differentiation regulation and in vivo applications, due to the diverse cell types and environments in vivo that are not accurately replicated in in vitro models.^89^ Thus, differentiation regulation in stem cell transplantation requires a comprehensive approach.

#### 4.2.3 Recruiting host cells and promote tissue integration

**FIGURE 7:**
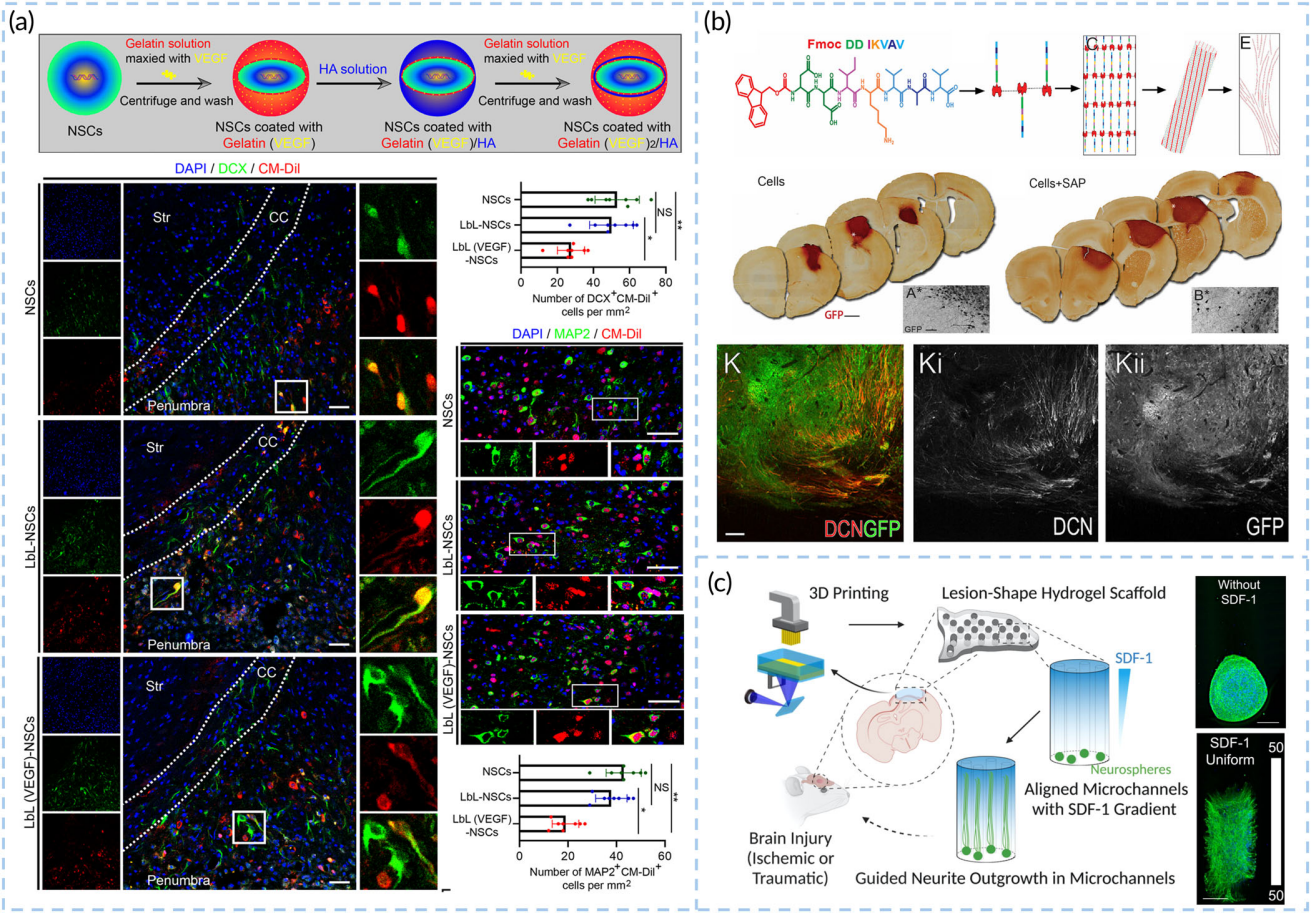
Biomaterials contribute to recruit host cells and promote tissue integration. (a) Engraftment of layer‐by‐layer (VEGF)‐NSCs reinforced neurogenesis, angiogenesis and promote endogenous DCX cells migration from LV to penumbra post‐dMCAO in mice. Reproduced with permission from Reference 137. (b) The IKVAV SAP scaffold was transplanted with NPCs in stroke cavity. Numerous GFP+ migrating neuroblasts, expressing doublecortin (DCX), were observed at the graft periphery and infiltrating into the host tissue. Reproduced with permission from Reference 107. (c) 3D printed the HA‐gelatin‐based scaffolds with spatial gradients of stromal‐derived factor 1 (SDF‐1), and it is able to effect on NSC migration. Reproduced with permission from Reference 99.

For transplanted cells to effectively contribute to repair, they must integrate seamlessly with host tissue. This requires both the migration of transplanted cells into surrounding tissue and the infiltration of host cells into the transplanted material. Intervention through the delivery of biological cues can facilitate this integration.^135^, ^136^, ^137^ For example, layer‐by‐layer (LbL) assembly of NSCs using gelatin and hyaluronic acid hydrogels, combined with VEGF loading, can promote the grafted NSCs proliferation and differentiation into neurons. At the same time, LbL (VEGF)‐NSCs reinforce neurogenesis, angiogenesis, and the survival of host neurons^138^ (Figure 7a). The self‐assembly of IKVAV peptide scaffold supported cell transplants and induced progressive motor improvements over 9 months. Numerous GFP+ migrating neuroblasts infiltrated into the host tissue^107^ (Figure 7b). During in situ delivery, biomaterials can serve as a “biological bridge,” supporting the integration of transplanted cells with host tissue. Artificial scaffolds containing laminin can mimic the vascular system, facilitating the formation and migration of neuroblast chains towards the damaged area.^139^ Shape‐specific hydrogels can guide oriented tissue regeneration. 3D‐printed hydrogels with microchannel designs that match the infarct shape and uniaxial alignment guide neurons to grow along the microchannels and create a gradient distribution of stromal‐derived factor 1 (SDF‐1), enhancing cell recruitment^99^ (Figure 7c).

#### 4.2.4 Modulating the microenvironment within the brain

**FIGURE 8:**
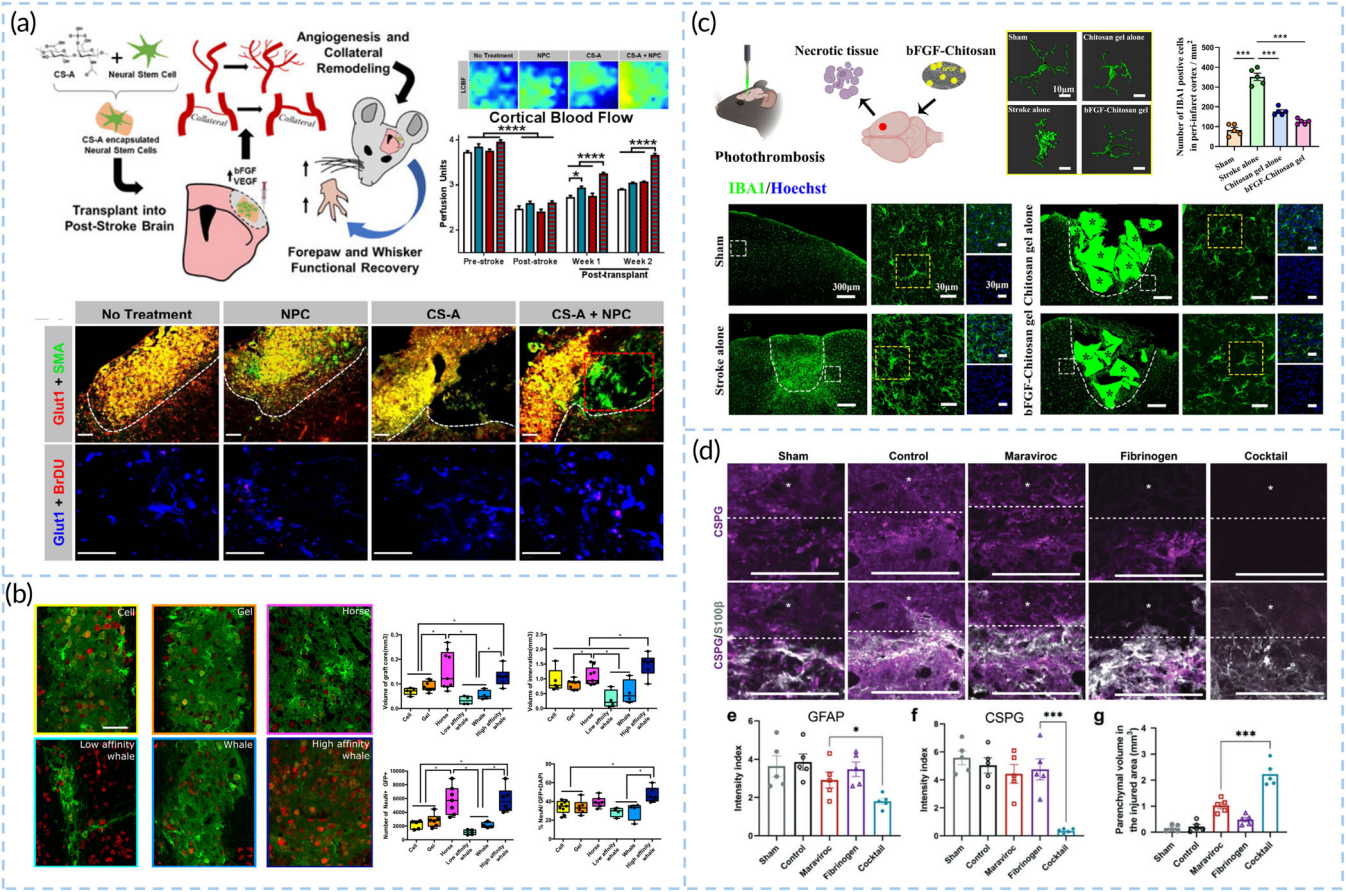
Biomaterials can modulate the microenvironment within the brain. (a) NPCs were encapsulated with a novel bFGF enriching chondroitin‐4‐sulfate A hydrogel and transplanted into the infarct core in mice. Treatment with encapsulated cells significantly enhanced angiogenesis, collateral vessel astrogenesis, blood flow, and functional recovery after ischemic stroke. Reproduced with permission from Reference 141. (b) Incorporation of myoglobin within SAPs based on Fmoc‐DDIKVAV improves graft NPCs survival and differentiation to neurons. Reproduced with permission from Reference 143. (c) bFGF‐Chitosan gel suppresses the activation of microglia in peri‐infarct area. Reproduced with permission from Reference 137. (d) A cocktail consisting of maraviroc, fibrinogen and NPCs was transplanted into stroke site. The expression of CSPG showed a significantly reduced level at the boundary of the cavity, indicating the reduction of glial scar. Reproduced with permission from Reference 118.

First, in stroke therapy, the reconstruction of blood supply in the infarct penumbra benefits neural regeneration and motor functional recovery^140^, ^141^, ^142^ (Figure 8a). The formation of new blood vessels is crucial for the migration and nourishment of neural cells.^138^ In addition to biological factors and drugs, hydrogels sensitive to MMPs can also promote angiogenesis.^117^ Given that transplanted cells often suffer from inadequate oxygen because of damaged vessels post‐IS, researchers have developed hybrid myoglobin‐peptide hydrogels to provide continuous oxygen delivery while offering physical and nutritional support to transplanted NPCs, positively affecting cell survival and differentiation^143^ (Figure 8b). Moreover, modulating the inflammatory cell phenotype is a key intervention target post‐stroke.^144^, ^145^, ^146^, ^147^ For example, bFGF‐Chitosan gel inhibits microglia activation in the peri‐infarct cortex of photothrombotic stroke mouse^137^ (Figure 8c). Chondroitin sulfate‐A hydrogels encapsulating NPCs, when implanted into the brain, promote a regenerative phenotype in microglia/macrophages through IL‐10 accumulation, enhancing angiogenesis and astrogenesis.^148^ A biopolymer hydrogel composed of cross‐linked hyaluronan and heparin sulfate promoted the survival of NPC lines in the infarct cavity by diminishing inflammatory infiltration of the graft with the hydrogel transplant.^149^ Just like pro‐inflammatory microglia, over‐activated astrocytes are detrimental to tissue recovery. Glial scars are a major barrier to the spread of transplanted cells; thus, biomaterials are also designed to reduce reactive astrocyte activity^118^, ^150^ (Figure 8d). Incorporating collagenase and trypsin into hyaluronic acid hydrogels may be an attractive strategy to facilitate cell migration.^151^

Materials provide stem cells with a simulated 3D in vivo environment, where adjusting the physical properties of the materials can influence cell behavior. The addition of biochemical signals further enhances the transplantation environment. Biomaterials can also improve the efficiency of cell reprogramming.^152^ In summary, while biomaterials serve as excellent multifunctional carriers for stem cells, there are currently limited preclinical studies (notably studies listed in Table 1); further investigation is needed.

**TABLE 1:** Experimental study in stem cell and biomaterials therapy for IS.

Authors, year	Cell type		Materials	Additional factors	Delivery time and route	Animal and model	Therapeutic effect	Ref.
Wang et al., 2023	NPC*		Fibrinogen gel	maraviroc	14 days post stroke, stroke cavity	SCID mice, PT*	Protects grafted NPCs from apoptosis, promote neural differentiation, mitigated glial reaction and restored vascularization, downregulates CCL and CCR5 expression	118
Ilan Vonderwalde et al., 2020		Directly reprogrammed NPC	Artificial cerebrospinal fluid or HA hydrogel		4 days post stroke, stroke site	Immunocompromised Fox Chase SCID/Beige mice, ET‐1 stroke	Promotes functional recovery, but NPC transplant survival is not necessary for recovery, increase perilesional expression of synaptophysin	74
Shin et al., 2018			Poly (glycolic acid)		7 days post stroke, stroke cavity	New burn ICR mice, Permanent right common carotid artery occlusion	Reduced the lesion volume, induced survival, engraftment, and differentiation of grafted cells, increased neovascularization, inhibited glial scar formation, altered the microglial/macrophage response	103
George et al., 2017	NPC		Polypyrrole	Electrical stimulation	7 days post stroke, cortex primarily on the penumbral cortex medial to the lesion	T‐cell deficient nude rats, dMCAO	Electrical stimulation changes RNA expression in hNPC (VEGFA pathway), enhance stroke recovery, increases peri‐infarct vasculature, alters rat cortical tissue	104
McCrary et al., 2020		Derived from iPSC	Chondroitin sulfate‐A hydrogel	bFGF*	7 days post stroke, stroke site	C57BL/6 male mice, dMCAO	CS‐A encapsulation significantly improves vascular remodeling, cortical blood flow, and sensorimotor behavioral outcomes	141
Lam et al., 2014	NSC*	Derived from iPSC	HA* hydrogel		7 days post stroke, stroke cavity	C57BL/6 male mice, PT	Mechanical properties of HA hydrogel can influence inflammatory response, promote the differentiation of cells	145
Liu et al., 2023			ECM* hydrogel	Matrix metallopeptidase (MMP)‐response	5 days post stroke, stroke cavity	C57BL/6 male mice, PT	Promoted iPSC‐NSC growth and differentiation on 2D surfaces and in 3D hydrogels, promoted iPSC‐NSC survival and differentiation in vivo	142
Zheng et al., 2021	NSC		N‐carboxyethyl chitosan and oxidized sodium alginate‐based polysaccharide hydrogel		1 day post stroke, stroke site	Sprague–Dawley rats, tMCAO	Support neurodegeneration in SVZ, inhibition of the astrocyte reaction and the increased expression of VEGF	150
Zhang et al., 2022			Hybrid hydrogel based on GelMA* and poly(3,4‐ethylenedioxythiophene): poly (styrene sulfonate)		5 days post stroke, stroke cavity	Sprague–Dawley rats, tMCAO	Promoted the development of NSCs into neurons in vitro, attenuated inflammatory responses in vivo	146
Liu et al., 2022			Viral nanofibers, electrostatically coated on biocompatible silk protein microparticles	RGD peptides	5 days post stroke, stroke cite	Sprague–Dawley rats, tMCAO	Induce NSCs differentiation in vitro, induce angiogenesis, limit inflammatory response, induce neurogenesis and functional recovery in vivo	115
Qi et al., 2022			Poly(trimethylene carbonate)15‐F127‐poly(trimethylene carbonate)15	BDNF*, NGF*, NT‐3*	14 days post stroke, stroke site	Sprague–Dawley rats, tMCAO	Reduced apoptosis, promoted differentiation of transplanted NSCs into mature neurons, reduce infarct size, improve neurological performance	121
Shabani et al., 2021	NSC		PLGA*‐PEG* micelle biomaterial	Reelin	7 days post stroke, stroke cavity	Balb/C mice, PT	Induce proliferation rate, neurite outgrowth and neuronal differentiation in vitro, increase the number of migrating neural progenitor cells and mature neurons, decreased the astrocytic gliosis, increased local angiogenesis, reduction of cavity size and promote neurological outcome	110
Lin et al., 2021			Superparamagnetic iron oxide nanoparticles and small interfering RNA/antisense oligonucleotides against Pnky lncRNA into NSCs		2 days post stroke, stroke cavity	C57BL/6 male mice, PT	Enhancement in neuronal differentiation in vitro and in vivo, no detrimental effect on migration capacity, reduced the lesion volume, improvement of neurologic function	152
Jiang et al., 2019			ROS‐responsive charge‐reversal poly[(2‐acryloyl)ethyl(p‐boronic acid benzyl) diethylammonium bromide as a gene transfection vector to make NSCs overexpress BDNF		C57BL/6, tMCAO	1 day post stroke, i.v. injection	BDNF‐NSCs home to ischemic regions, lead to augmented BDNF levels, enhances the mouse survival rate	153
Bernstock et al., 2019	NSC		/	Engineer NSCs via upregulation of global SUMOylation	C57BL/6J, tMCAO*	3 days post stroke, intraparenchymal	Resistance to OGD/ROG and enhanced neuronal differentiation in vitro, increased survival and neuronal differentiation in vivo	63
Wang et al., 2020			/	NSCs were transduced with circHIPK2 siRNA	C57BL/6J, tMCAO	7 days post stoke, contralateral lateral ventricle	Silencing of circHIPK2 facilitated differentiated to neurons in vitro, increased neuronal plasticity, conferred long‐lasting neuroprotection, reduced functional deficit in vivo	160
Jiang et al., 2022		NSCs‐extracellular vesicles	Phenylboronic acid‐modified hyaluronic acid (HA) and polyvinyl alcohol	Phenylboronic acid derivatives: glucose responsiveness and ROS scavenging properties	Cortex of the infarcted hemisphere	Type 2 diabetes mellitus mice, dMCAO*	Reduced brain atrophy volume, increase angiogenesis	159
Somaa et al., 2017	ESC*‐cortical progenitor		Peptide‐based scaffolds (IKVAV)		Twice: 6 days or 3 weeks after stroke, stroke site	Athymic (CBHrnu) nude rats, ET‐1 stroke	Promoted graft maturation and integration, reduced host tissue atrophy, resulting in improved motor function over a period of 9 months	107
Fernández‐García et al., 2018	BMSC*		Chitosan‐collagen scaffold		3 days post stroke, Caudate putamen striatum of the infarcted hemisphere	CD‐1 mice, dMCAO*	Attenuate the cerebral damage, induce a delayed cortical plasticity in the peri‐lesional tissue	158
Zhang et al., 2017			Gel‐like scaffold from plasma		3 weeks post stroke, stroke cavity	Sprague–Dawley rats, tMCAO	Reduce infarct lesion, improve motor function	158
Kang et al., 2024			Gelatin‐Hydroxyphenyl hydrogels		7 days post stroke, temporalis muscle/cerebrum interface	Sprague–Dawley rats, Chronic cerebral hypoperfusion model	Promoting neovascularization, facilitating neuronal differentiation, suppressing neuroinflammation	147
Zamproni et al., 2019			PLA polymeric rough microfibrous scaffolds		3 days post stroke, stroke site	C57BL/6 male mice, thermocoagulation in the submeningeal blood vessels	MSC transplantation decreases the lesion size and the scaffold increases MSC retention	98
Jiang et al., 2024	GMSC*‐derived neural lineage cells		HA hydrogel		8 days post stroke, stroke site	Sprague–Dawley rats, tMCAO*	Facilitate the proliferation and differentiation of GMSCs‐derived neural lineage cells.	120
Wang et al., 2022	BDNF‐overexpressing MSC*		Thiolated gelatin, Thiolated HA, and polyethylene glycol diacrylate		3 days post stroke, stroke cavity	C57BL/6 male mice, PT	Improved functional recovery, neurogenesis, white matter recovery, and angiogenesis	54
Sideris et al., 2022	–		HA based microporous annealed particle (MAP)	/	5 days post stroke, stroke cavity	C57BL/6 male mice, PT*	Modulate inflammation by reducing the number of reactive neurotoxic astrocytes and preventing influx of reactive microglia/macrophages	144
Nih et al., 2018	–		In situ gelling HA hydrogel	VEGF*	5 days post stroke, stroke cavity	C57BL/6 male mice, MCAO and PT	Promote neurological recovery, promote vascular ingrowth into infarct cavity and accompanying axonal network	135
Liu et al., 2022	–		RADA16‐I	Cerebral dopamine neurotrophic factor (CDNF*)	3 days post stroke, lateral ventricle	Sprague–Dawley rats, tMCAO	Neuroprotective effect, promotes the proliferation and neuronal differentiation of cultured NSCs and ischemia rats	161
Damian et al., 2021	–		Extracellular matrix (ECM) hydrogel	–	7, 14, 28, 90 days post stroke, stroke cavity	Sprague–Dawley rats, tMCAO	Implantation timing affects biodegradation, host cell infiltration, vascularization	162
Ghuman et al., 2018	–		Porcine‐derived urinary bladder matrix (UBM)‐ECM hydrogel	–	14 days post stroke, ventral posterior region of the cavity	Sprague–Dawley rats, tMCAO	Weaker ECM hydrogels undergo efficient biodegradation, reduce tissue cavitation, promote endogenous cell invasion and neovascularization	105
Duan et al., 2023	–		Chitosan gel	bFGF	7 days post stroke, stroke cavity	Wistar rats, PT	Activates endogenous neural stem/progenitor cells proliferation, migration and maturation, facilitates angiogenesis	88

Abbreviations: NPC, neural precursor cell; NSC, neural stem cell; MSC, mesenchymal stem cell; BMSC, bone marrow mesenchymal stem cell; iPSC, induced pluripotent stem cell; ESC, embryonic stem cell; HA, hyaluronic acid; ECM, extracellular matrix; PLA, polylactic acid; PLGA, polylactic‐co‐glycolic; PEG, polyethylene glycol; bFGF, basic fibroblast growth factor; BDNF, brain‐derived neurotrophic factor; NGF, nerve growth factor; NT‐3, neurotrophin‐3; PT, photothrombotic model; tMCAO, transient middle cerebral artery occlusion; tMCAO, distal middle cerebral artery occlusion.

## 5 CLINICAL TRIALS OF BIOMATERIAL‐BASED STEM CELL THERAPY

### 5.1 Current status of clinical trials

Basic research on stem cell therapy for ischemic stroke has yielded encouraging results. Preliminary clinical studies have demonstrated the safety of stem cell transplantation. However, some studies have reported adverse reactions, including allergic reactions and infections.^154^, ^155^ Although numerous clinical trials have utilized functional assessment scales such as the NIHSS (National Institutes of Health Stroke Scale), BI (Barthel Index), Ashworth Scale, and FM (Fugl‐Meyer Assessment), no significant functional recovery advantage has been observed in patients receiving stem cell transplantation; one trial noted that BMCS transplantation reduced patient mortality after 5 years, suggesting that efficacy may require long‐term observation.^156^

In the infarct cavity of adult male macaques following ischemic stroke, combined injection of hyaluronic acid hydrogel and BDNF was administered. Two weeks later, significantly higher levels of BDNF were detected at a distance of 2 cm from the infarct site compared to the group receiving BDNF alone.^157^ This study suggests that in environments more akin to the human brain, biomaterials facilitate the sustained release of growth factors. Despite this, no biomaterials have been approved for stroke clinical trials.

We attempted to identify clinical trials with objectives similar to ours (Table 2). Encouragingly, a Phase I/II clinical trial in Germany was designed for implantation into the cavity after intracerebral hemorrhage of microcapsules containing allogenic MSCs (GLP‐1 CellBeads, NCT01298830). The objective of this study is to assess the safety of GLP‐1 CellBeads® in patients with space‐occupying intracerebral hemorrhage. A clinical trial in China (NCT02767817) attempts to stereotactic intracranial hematoma and insert the drainage tube to inhale hematoma after brain injury for 1–2 days; then injectable collagen scaffold combined with 10 million MSCs were transplanted into the hematoma cavity before the drainage tube was pulled out. RGTA® (ReGeneraTing Agent, NCT04083001) is a synthetic polysaccharide mimicking the extra‐cellular matrix scaffold. The study aims to evaluate the safety and tolerability of a single intra‐arterial injection of RGTA® in acute ischemic stroke patients. The aforementioned studies primarily investigate the safety profile of biomaterial and cell transplantation in cerebral injury. Despite this, no clinical trials have conclusively demonstrated the therapeutic efficacy of biomaterial‐assisted cell therapy for cerebral ischemia. Turning to spinal cord injury—another central nervous system disorder—we see similar clinical research progress in repair strategies. NeuroRegen Scaffold™ is a collagen‐based hydrogel for chronic spinal cord injury. Two finished clinical trials (NCT02688049, NCT02352077) have proved that NeuroRegen scaffolds™ loaded with MSCs are safe and improve partial shallow sensory and autonomic nervous function. Another clinical trial conducted in China has explored the combined use of stromal vascular fraction (SVF) and self‐assembling peptide nanofiber hydrogel for the treatment of traumatic spinal cord injury (NCT05967325). SVF is a heterogeneous mixture of cells obtained from adipose tissue, including adipose‐derived stem cells, endothelial cells, and immune cells. While pathological differences exist between spinal cord injury and cerebral ischemia, these studies nevertheless provide valuable insights for repair strategies, particularly in white matter regeneration.

**TABLE 2:** Clinical trials on combined therapy of biomaterials and stem cells.

NCT number	Title	Country	Study started	Status	Stem cells	Biomaterials	Condition
NCT01298830	GLP‐1 CellBeads® for the Treatment of Stroke Patients with Space‐occupying Intracerebral Hemorrhage	Germany	2008‐10	Phase 1 Phase 2	Allogenic mesenchymal cells, transfected to secrete Glucagon like peptide‐1	Alginate microcapsules	Intracerebral hemorrhage
NCT02767817	Injectable Collagen Scaffold™ Combined with MSCs Transplantation for Brain Injury	China	2016‐03	–	Mesenchymal cells	Collagen scaffold	Brain injury
NCT04083001	An Open, Study to Assess the Safety of RGTA® (OTR4132) in Patients with Acute Ischemic Stroke (AIS) (MATRISS)	France	2022‐03	–	–	RGTA® (Heparan sulphates)	Acute ischemic stroke
NCT02352077	NeuroRegen Scaffold™ With Stem Cells for Chronic Spinal Cord Injury Repair	China	2015‐01	Phase 1	Bone marrow mononuclear cells or mesenchymal stem cells	NeuroRegen Scaffold™ (collagen scaffold with multiple functional nerve regeneration molecules)	Spinal cord injury
NCT02688049	NeuroRegen Scaffold™ Combined With Stem Cells for Chronic Spinal Cord Injury Repair	China	2016‐02	Phase 1 Phase 2	Mesenchymal stem cells or neural stem cells	NeuroRegen Scaffold™	Spinal cord injury
NCT05967325	SVF Combined with Functional Self‐assembling Peptide Nanofiber Hydrogels in the Treatment of Spinal Cord Injury	China	2023‐07	–	Stromal vascular fraction (include adipose‐derived stem cells, endothelial cells, endothelial progenitor cells, pericytes, T cells, and other immune cells)	Self‐assembling peptide nanofiber hydrogels (HGF(RADA)4RIKVAV)	Spinal cord injury

### 5.2 Challenges in clinical trials

The clinical translation of stem cell therapies faces numerous challenges. Immune rejection remains a critical issue that cannot be circumvented.^144^ Compared to allogeneic cells, autologous cells exhibit lower immunogenicity and reduced risk of allergic reactions post‐implantation. However, allogeneic cells are more amenable to large‐scale production and standardized storage. During cell production, variations in preparation methods and equipment may lead to differences in molecular phenotypes, highlighting the challenges in quality control of cell‐based products.^67^ In addition, cells with strong self‐renewal potential may pose a risk of tumor formation post‐transplantation.^139^ Although in mouse studies, researchers have utilized iPSCs carrying the suicide gene thymidine kinase (activated by Ganciclovir) for intracranial delivery, functionalized hydrogels were shown to prolong GCV presentation, thereby reducing iPSC‐derived teratoma formation and proliferating cells in neural grafts.^55^ However, such methods remain distant from clinical translation, particularly due to challenges in ethical approval. The formation of scar tissue by transplanted cells is another adverse effect. Studies have found that bone marrow‐derived mononuclear cells, when intravenously administered during the subacute phase after stroke, may form scar tissue in the brains of patients.^59^ Similarly, these issues may potentially be mitigated in the future through the co‐transplantation of biomaterials.

Except for those listed in section 5.1 there are no more clinical trials related to biomaterial‐assisted stem cell treatment for IS. Even studies based on large animals are insufficient. While current clinical trials primarily establish the safety profile of biomaterial transplantation, future studies should choose scaffolds with enhanced cellular affinity once safety is met. Furthermore, the ischemic cavities in human patients exhibit significantly larger volumes than rodent lesion models, necessitating exponentially greater biomaterial quantities for complete filling. Thus, future clinical applications face difficulties in controlling the spatial distribution of implanted scaffolds. Controlling and monitoring cellular distribution in scaffolds also remains a challenge. The development of non‐invasive imaging modalities is imperative to optimize and monitor stem cell‐biomaterial delivery and therapeutic efficacy. Clinical translation of biomaterials as cell carriers must consider the following: (1) The hardness of the biomaterial must match brain tissue to avoid new space‐occupying risks, and it should be degradable with harmless degradation products and within a safe swelling rate. (2) Not all stroke patients are suitable for biomaterial transplantation. Factors such as lesion location, severity, and patient age need extensive clinical trials to determine suitability. (3) Biomaterials must be cost‐effective and amenable to standardized production to avoid expensive and lengthy clinical approvals. (4) Post‐transplant evaluation methods need further research to ascertain whether exogenous cells truly compensate for the lost neural circuits.

## 6 SUMMARY AND OUTLOOK

The complexity of the nervous system and the suboptimal prognosis of ischemic stroke patients have created an urgent need for novel therapeutic approaches. Stem cell transplantation has achieved measurable progress in stroke therapeutics. Biomaterials have opened new horizons for stem cell therapy, serving as a delivery platform that may make localized stem cell transplantation a clinical reality. Rational biomaterial design improves stem cell therapeutic outcomes. The clinical translation of combined biomaterial‐stem cell therapies for IS continues to face multifaceted challenges. Although the complexity and specificity of biomaterials are continually improving, achieving an effective balance between efficacy and translational potential remains difficult. Encouragingly, pioneering clinical trials have begun to emerge. We believe that strengthened collaboration between research institutions and clinical centers will accelerate the progression to large‐scale clinical trials. The integration of these novel therapeutic approaches with conventional neural rehabilitation strategies holds significant potential to substantially improve functional recovery and promote quality of life for IS patients.

## AUTHOR CONTRIBUTIONS

M.W. and Y.R. performed the literature search and wrote the original draft. Z.L., L.Y., and W.S. designed, critically revised, and supervised the manuscript. J.L., F.L., and Z.D. collected relevant information and created the figures. N.L. and J.X. designed the tables. All the authors discussed and commented on the whole article. All authors have read and agreed to the published version of the manuscript.

## CONFLICT OF INTEREST STATEMENT

The authors declare no conflicts of interest.

## Data Availability

Data sharing not applicable to this article as no datasets were generated or analysed during the current study.
